# Supramolecular peptide constructed by molecular Lego allowing programmable self-assembly for photodynamic therapy

**DOI:** 10.1038/s41467-019-10385-9

**Published:** 2019-06-03

**Authors:** Huangtianzhi Zhu, Huanhuan Wang, Bingbing Shi, Liqing Shangguan, Weijun Tong, Guocan Yu, Zhengwei Mao, Feihe Huang

**Affiliations:** 1 State Key Laboratory of Chemical Engineering, Center for Chemistry of High-Performance & Novel Materials, Department of Chemistry, Zhejiang University, 310027 Hangzhou, P. R. China; 2 MOE Key Laboratory of Macromolecular Synthesis and Functionalization, Department of Polymer Science and Engineering, Zhejiang University, 310027 Hangzhou, P. R. China; 3 Laboratory of Molecular Imaging and Nanomedicine, National Institute of Biomedical Imaging and Bioengineering, National Institutes of Health, Bethesda, MD 20892 USA

**Keywords:** Self-assembly, Self-assembly, Self-assembly

## Abstract

Peptide self-assemblies with multiple nanostructures have great potentials in functional biomaterials, and yet the tedious and costly covalent peptide modification and the lack of facile controllability on self-assembly morphology retard the peptide-related exploration. Here we report a simple approach to fabricate a supramolecular peptide that shows programmable self-assembly with multiple morphologies and application in photodynamic therapy. Pillar[5]arene-based host−guest recognition is used to construct a supramolecular peptide, which simplify the peptide modification and promote the controllability of the self-assembly behavior. Due to the ERGDS sequences on the exterior surfaces and hydrophobic cores of self-assemblies, the nanoparticles formed from the supramolecular peptide are suitable vehicles to encapsulate a photosensitizer for photodynamic therapy. In vitro and in vivo studies demonstrate that the inherent targeting capability and supramolecular strategy greatly boost its photodynamic therapeutic efficiency. This supramolecular peptide holds promising potentials in precise cancer therapy and perspectives for the peptide modification.

## Abstract (web summary)

Despite its application in functional materials, covalent peptide modification and controlling peptide self-assembly remains challenging. Here the authors report a programmable supramolecular peptide by pillararene-based noncovalent interactions with multiple self-assembly morphologies and application in PDT.

## Introduction

With distinguished advantages such as high biocompatibility, cell permeability, and low immunogenicity, peptides are favorable candidates for functional materials at the nano- or macroscale in the biochemical field^1–3^. The variety of combinations by 20 natural amino acids renders peptide-based materials with high diversities, including hydrogels for extracellular matrix, hybrid materials for biosensing, and catalysis^4–6^. Particularly appealing cases are well-defined nanostructures self-assembled by peptide amphiphiles because the hydrogen bonds between peptides render the self-assemblies well-defined and the functional heading groups located on the exterior facilitate their bio-applications^7–9^. Depending on peptide sequences, physical conditions and external stimuli, peptide amphiphiles are able to self-assemble into fibers, nanotubes, vesicles, and nanoparticles (NPs), and such assemblies have been applied in drug delivery, cell imaging, tissue engineering, and membrane protein stabilization^10–14^. Two main points are focused on long-term applications of these materials: chemical modifications of peptides and stabilities of self-assemblies in different conditions^15^. In order to achieve specific functions or controllable self-assembly morphologies, tedious covalent modifications and purifications of peptides are required because the self-assembly behaviors of unmodified peptides hardly match the requirements^16,17^. Additionally, the ways to enhance the stability of self-assemblies usually involve covalent attachments or co-assemblies with synthetic polymers^18,19^, which benefit from the robustness of synthetic polymers but suffer from partially altered functionalities of peptides^20^. Therefore, to expand the potential of peptide-based materials, it’s urgent to find convenient methods to control the self-assembly process and to increase the stability of the self-assembly without the help of synthetic polymers or tedious covalent modifications, which encourages us to explore the fabrication of supramolecular peptides. For this kind of fabrication, the modification of the peptides is dominant by non-covalent interactions and it is unncessary to do covalent synthesis and/or purification.

Pillararene-based supra-amphiphiles, which bridge the gap between controllable self-assembly and simple synthesis by non-covalent interactions, are being actively investigated^21–23^. Compared with other macrocycles, facile functionalization, reliable host−guest recognition and multiple stimuli-responsivenesses of pillararenes endow the supra-amphiphiles with various morphologies and properties such as vesicles for controlled release and NPs for cell imaging^24–30^. Therefore, incorporation of pillararenes into peptide assemblies is a promising approach to modify the self-assembly behavior and further promote the function^31^.

Herein, we develop a facile strategy to construct a supramolecular peptide, of which the self-assembly behavior can be controlled by pillararene-based host−guest interactions and temperature. Compared with traditional covalent bond-involved peptide modification, such supramolecular peptide doesn’t require any purification and shows exciting advantages in simple preparation, stimuli-responsiveness, and controllability. Besides, the functionalities of the peptide such as biocompatiblity and target ability greatly maintain after the non-covalent modification, which offers a facile approach to construct peptide-involved biomaterials. Interestingly, NPs self-assembled from the supramolecular peptide act as excellent nanovehicles to stably encapsulate a photosensitizer, boosting the photosensitizing effect to effectively ablate the tumors.

## Results

### Design principles of the peptide sequence and the supramolecular peptide

**Fig. 1:**
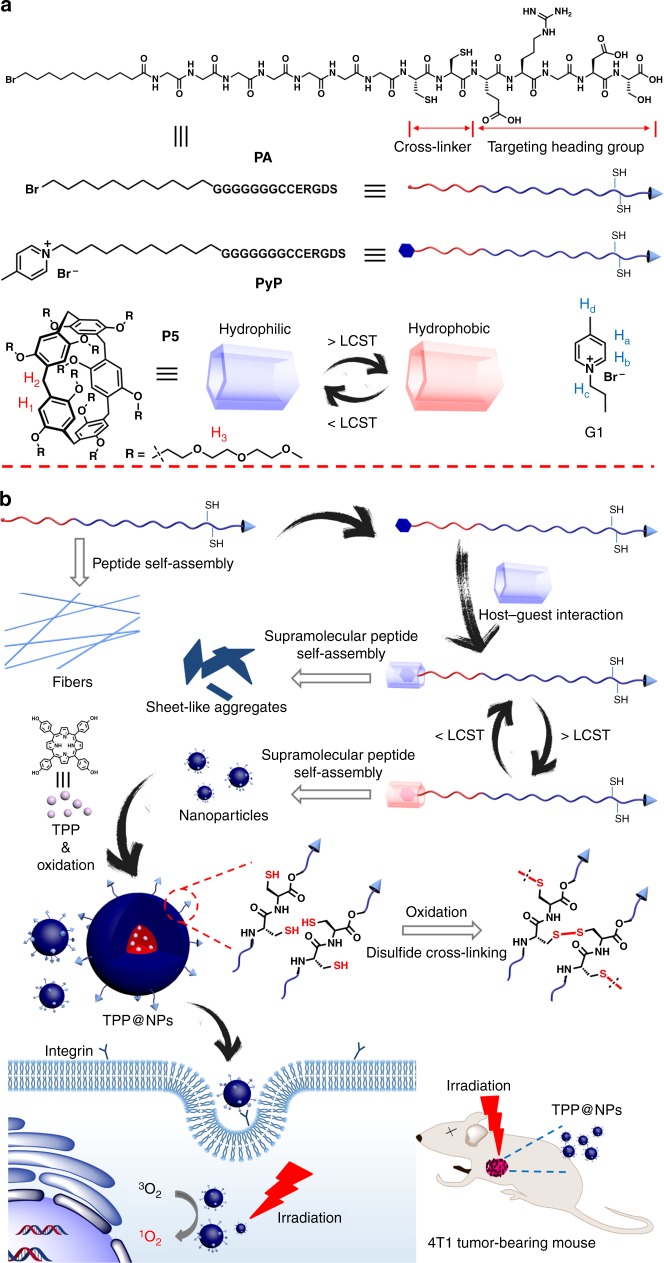
Design and construction schematics of the supramolecular peptide. **a** Chemical structures and cartoon representations of PA, PyP, and P5. **b** Schematic illustrations of the programmable peptide self-assembly and PDT process. Solid arrows indicate experimental procedure and hollow arrows refer to self-assemblies or structural details

The peptide sequence (Fig. 1a, G_7_CCERGDS, G = glycine, C = cysteine, E = glutamic acid, R = arginine, D = aspartic acid, S = serine) was designed as follows: (1) multiple hydrogen bonding close to the hydrophobic core played an indispensable role in forming the nanostructure by self-assembly, and thus seven glycine moieties were used to form strong multiple hydrogen bonds;^32^ (2) cysteine units were easily oxidized to form disulfide cross-linkers by air, endowing the self-assembly with structural integrity even after the external stimulus is removed, which is crucial to maintain the functionalities of the peptide as well as its assembly;^33,34^ (3) the ERGDS heading group promoted the cellular internalization of the self-assembled NPs by cancer cells overexpressing *α*_v_*β*_3_ integrin through receptor-mediated endocytosis^35^. 11-Bromo-alkyl group at *N*-terminal performed as a hydrophobic component during the self-assembly process. More importantly, the bromine atom could be easily substituted by 4-methylpyridine to generate a cationic guest unit for pillar[5]arene^36,37^. A water-soluble pillar[5]arene bearing ten tri(ethyleneoxide) groups (P5) is hydrophilic at room temperature and gradually becomes hydrophobic as temperature rises^38,39^. Thus, P5 was used in this work due to its unique thermo-responsive property and strong binding affinity to cationic guests, which is accompanied by the main advantage that the thermo-responsive group could be easily attached to the peptide to control the self-assembly behavior without tedious covalent synthesis. The accurate and reliable combination of P5 and PyP is similar to Lego blocks, and these molecular Lego were able to aggregate to assemblies including sheets and NPs with facile controllability. The resultant NPs with targeting ligands on the surfaces were suitable carriers for a hydrophobic photosensitizer to enhance its PDT efficiency (Fig. 1b).

### Study of the host−guest complexation and thermo-responsiveness

**Fig. 2:**
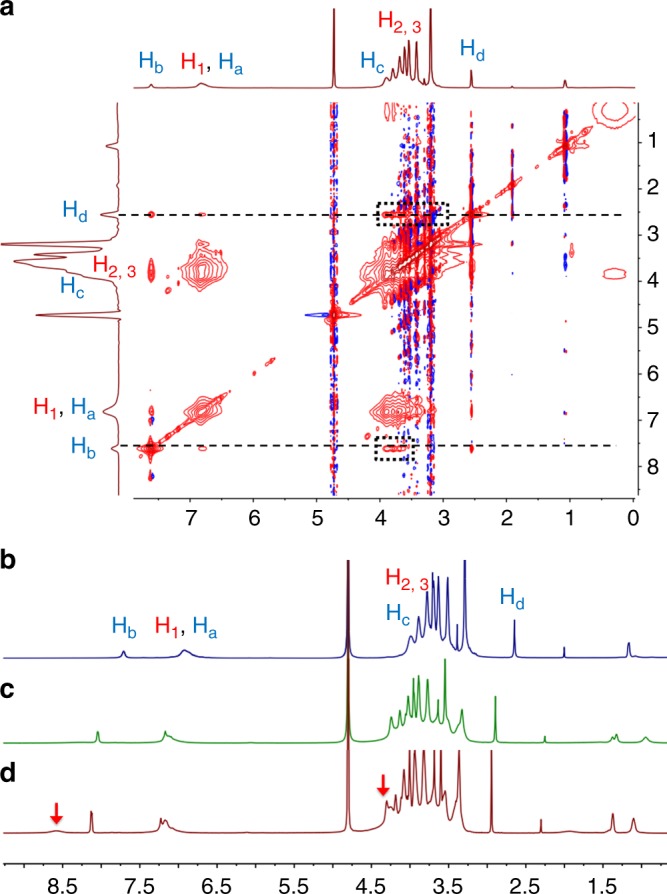
Study of host−guest interactions at various temperatures. **a** 2D NOESY spectrum (400 MHz, D_2_O) of G1 + P5 (5.00 mM). The correlation signals between the host and guest were marked with rectangles. ^1^H NMR spectra (400 MHz, D_2_O) of G1 + P5 (5 mM) at **b** 25 °C, **c** 45 °C, and **d** 50 °C. Peaks belonging to de-threaded guest are marked with red arrows in **c**

Before studying the self-assembly behaviors, we first explored the host−guest recognition between P5 and a model guest (G1) by ^1^H NMR. Upon the addition of P5 (1.0 equiv.) to a solution of G1 (2.0 mM in D_2_O), the peaks related to H_a_, H_b_, and H_c_ on G1 shifted upfield. Strong NOE correlation signals between protons H_2_ and H_3_ on P5 and protons H_b_ and H_d_ on G1 were observed in a 2D NOESY spectrum (Fig. 2a and Supplementary Fig. 8). These results indicated that G1 could be complexed by P5 at room temperature. The association constant (*K*_a_) was determined to be 5.7 × 10^5^ M^‒1^ by isothermal titration calorimetry (ITC) in a 1:1 complexation mode (Supplementary Fig. 9). Besides, ^1^H NMR experiments at various temperatures were carried out to study the stability of host−guest complex (Fig. 2b, c, and d and Supplementary Fig. 10a). When a solution of host/guest mixture was heated to 45 °C, the host−guest complex was still stable. However, the peaks related to the de-threaded guest appeared when the temperature rose to 50 °C and these peaks became obvious and sharp at 60 °C, indicating the dissociation of the host−guest complex. Therefore, the complexation could be maintained lower than 50 °C, which was crucial to our thermo-responsive system.

Next, we examined the lower critical solution temperature (LCST) of the host−guest complex. Since the hydrophilic tri(ethyleneoxide) groups could turn to be hydrophobic by heating, P5 became hydrophobic and preferred to aggregate when the temperature was higher than the LCST, leading the solution opaque. Therefore, the transmittance of the solution at 700 nm was used to measure the cloud point (*T*_cloud_, Supplementary Fig. 10b). Upon heating the P5 solution, *T*_cloud_ of P5 was determined to be 42 °C. In presence of G1, *T*_cloud_ of the host−guest complex increased to 48 °C. When the host−guest complex was dissolved in PBS rather than pure water, the *T*_cloud_ of the complex decreased to 43 °C. This phenomenon that was also found in other thermo-responsive systems ascribed to the increase of the ion concentration in PBS^40,41^. Notably, *T*_cloud_ of the host−guest complex in PBS was lower than 50 °C, which meant the hydrophilicity of the complex could be adjusted without hampering the host−guest recognition.

### Fabrication of a supramolecular peptide and its programmable self-assembly

**Fig. 3:**
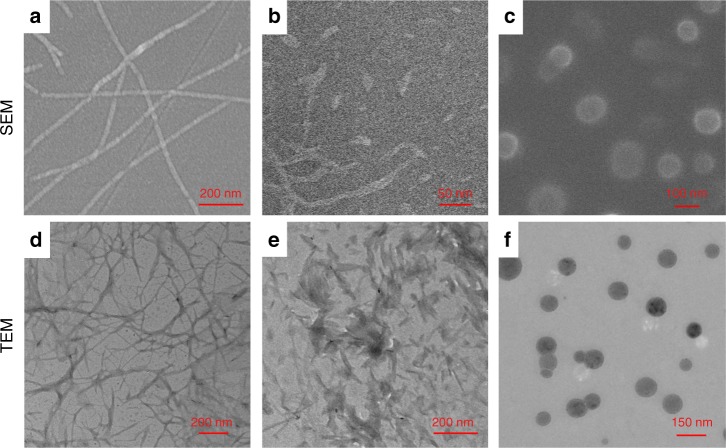
Programmable supramolecular peptide self-assembly process. SEM and TEM images: **a,** **d** PA; **b**, **e** P5 + PyP at 25 °C; **c,** **f** P5 + PyP at 45 °C

After the establishment of the host−guest recognition and the thermo-responsive behaviors, we investigated whether we could control the peptide self-assembly by host−guest interactions and temperature changes. To eliminate the repelling force generated by the negative charges on glutamic acid, aspartic acid and serine, 2.00 molar equivalent of Ca^2+^ were added to initiate the self-assembly process. The morphologies and sizes of the self-assemblies were subsequently studied by scanning electron microscopy (SEM) and transmission electron microscopy (TEM). As shown in Figs. 3a and 2d, PA self-assembled to nanofibers in the presence of Ca^2+^ with a uniformly distributed width of ~15 nm because of the hydrogen bonds between glycine units close to the hydrophobic alkyl chain. After the bromide atom was substituted by the pyridinium cation (PyP), nanofibers disappeared and no obvious assemblies were found, which was probably due to the loss of amphiphilicity. When a molar equivalent of P5 was added to the solution of PyP, irregular sheet-like assemblies with varying sizes appeared along with the formation of the host−guest complex (Fig. 3b and e). The formation of these assemblies was collaboratively driven by the π–π interactions between pillararenes and hydrogen bonds between peptides. Interestingly, the irregular assemblies changed into NPs with diameters of ~120 nm upon heating the solution of P5 and PyP to 45 °C in an air-flow oven (Fig. 3c and f). The average diameters of these NPs were also determined by dynamic light scattering (DLS, Supplementary Fig. 11), which was 135 nm. The morphology transformation was explained by the change of hydrophilicity of P5. Upon heating to 45 °C in PBS, water-soluble P5 became hydrophobic, leading to the formation of a supramolecular peptide with a hydrophilic peptide tail, which further aggregated into NPs by turning the hydrophilic peptide tail towards water and hydrophobic P5 and alkyl chain towards the inside of the NPs. Moreover, cysteine units on the peptide were sensitive to oxygen, and thus they were oxidized to cystine upon heating in air. The oxidation process rendered NPs stable by forming a disulfide cross-linked polymeric shell. According to the TEM images of the self-assemblies stored at 37 °C for one day, most of the NPs still remained even though P5 became hydrophilic. From these results, although the preparation of NPs required heating, once NPs formed, they were stable enough in a certain period at 37 °C for further biological experiments.

We also tested the stability of NPs under reduction conditions. Glutathione (GSH) and tris(2-carboxyethyl)phosphine (TCEP) were chosen as reducing agents to reduce disulfide bonds in NPs. After 3.0 molar equivalent of reducing agent was added to NPs (10.0 mM), the mixture was shaken at room temperature for 2 h and then dropped on the copper matrix and frozen dried. From the TEM images (Supplementary Fig. 12), upon addition of GSH, the integrity of NPs changed obviously along with the decrease of the size resulting from the cleavage of disulfide bonds in NPs. Treatment with TCEP induced the morphology transformation to sheet-like aggregates with higher efficiency attributed to higher reducing activity of TCEP. These results indicated the disulfide bond played an indispensable role in stabilizing NPs. GSH is overexpressed in cancer cells, but the high level of intracellular GSH can deplete the generated ^1^O_2_, thus dramatically weakening the PDT efficiency. The partial collapse of NPs induced by GSH demonstrated that NPs were potentially able to consume GSH, which was crucial for enhancing the PDT efficiency by a synergistic effect.

### Encapsulation of photosensitizer and singlet oxygen generation

From the studies above, the self-assembly morphology of the peptide was easily controlled by the P5 and heat, which encouraged us to explore their potential applications. Considering the exterior surfaces of the NPs were ERGDS-containing peptide chains, these NPs could be internalized by cancer cells overexpressing integrins (such as A549 cells). Besides, the interior regions of the NPs were hydrophobic upon heating, and could be used as carriers for hydrophobic species such as tetrakis(4-hydroxyphenyl)porphyrin (TPP) for PDT.

To encapsulate TPP, a reprecipitation method was used by adding a mixture of **PyP**/**P5**/TPP in acetone/water (1:2, 0.9 mL) to PBS (10 mL) at 45 °C under an oxygen atmosphere, and then acetone and excess TPP were removed by dialysis. As a result, a homogeneous suspension containing only a little TPP precipitate was obtained for the TPP@NPs assemblies with a loading efficiency of 65% (calculated according to a standard curve of UV absorbance of TPP versus concentration, Supplementary Fig. 13), whereas an obvious purple precipitate was observed for free TPP in PBS. UV absorption was subsequently measured (Supplementary Fig. 14), which suggested that TPP@NPs exhibited a higher and tiny blue-shifted (~2 nm) Soret band than that of free TPP in PBS, indicating that the π–π stacking between TPP was suppressed in the core of TPP@NPs. The weakened π–π stacking was also confirmed by fluorescence spectroscope. The solution of TPP@NPs showed a stronger fluorescence than that of TPP in PBS (Supplementary Fig. 15). As the strong π–π stacking between TPP inhibited its photoactivity, the ability to generate singlet oxygen (^1^O_2_) probably was recovered after encapsulation of the photosensitizer in NPs. Consequently, we tested the generation of singlet oxygen by detecting the fluorescent intensity of 1,3-diphenylisobenzofuran (DPBF) in presence of TPP@NPs^42,43^. DPBF emitted strong fluorescence, and yet the fluorescence decreased when DPBF underwent an oxidative degradation to form 1,2-dibenzoylbenzene. From the fluorescence spectra of the mixture of DPBF and TPP@NPs in PBS, the reduced fluorescent intensity of DPBF at 470 nm was observed upon irradiation with 660 nm laser (300 mW cm^–2^). After irradiation for 5 min, the fluorescent intensity of DPBF reduced to 57.5%, which clearly indicated its oxidative decomposition. However, less DPBF was oxidized in free TPP suspension. These results meant that the encapsulation of TPP in NPs enhanced its photosensitizing effect, facilitating to improve the photodynamic effect (Supplementary Fig. 16).

### Cellular uptake of TPP@NPs

**Fig. 4:**
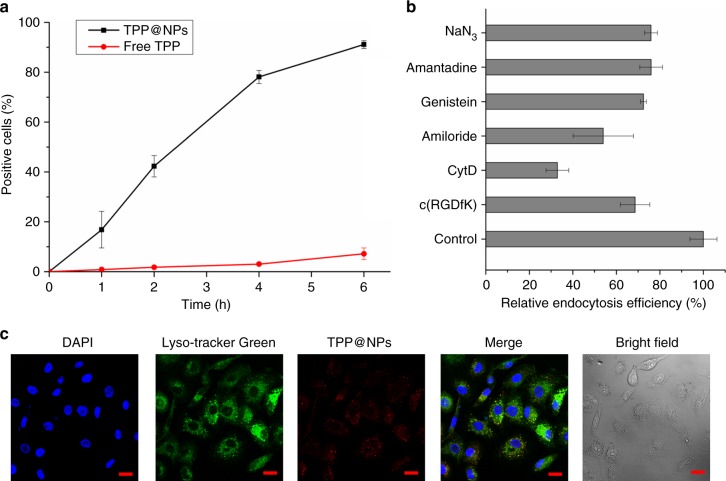
Cellular uptake of TPP@NPs. **a** Time-dependent cellular uptake of free TPP (red line) and TPP@NPs (black line). **b** Influence of pharmacological inhibitors on cellular uptake of TPP@NPs by A549 cells. Concentration of inhibitors: amantadine HCl, 1.00 × 10^‒3^ mM; genistein, 100 mM; amiloride HCl, 2.00 mM, CytD, 10.0 μg mL^‒1^; c(RGDfK), 50.0 nM. **c** 2D CLSM images of A549 cells stained by TPP@NPs (red), Lyso-tracker Green (staining lysosomes, green) and DAPI (staining nucleus, blue). The scale bar was 20 μm. Data are expressed as means ± s.d. (*n* = 3)

After encapsulating TPP in NPs, we investigated the cellular uptake of TPP@NPs by flow cytrometry (Fig. 4a). A549 cells were incubated with TPP@NPs and free TPP, respectively. From the time-dependent endocytosis, TPP@NPs showed enhanced endocytosis efficiency compared with free TPP. This is ascribed to the proper size, ERGDS sequences of NPs and low solubility and weak fluorescence of free TPP. Moreover, to study the possible endocytosis pathways of TPP@NPs, endocytosis inhibitors including sodium azide (decreasing cellular respiration), amantadine hydrogen chloride (inhibitor of clathrin-mediated endocytosis), genistein (inhibitor of caveolae-mediated endocytosis), amiloride hydrogen chloride (inhibitor of macropinocytosis), CytD (inhibitor of cytoskeleton), and c(RGDfK) (competitor of ERGDS sequences on TPP@NPs) were used to pretreat A549 cells. The pretreated A549 cells were incubated with TPP@NPs for another 6 h, and the uptake of TPP@NPs or free TPP was analyzed by flow cytometry. According to Fig. 4b, the endocytosis of TPP@NPs were possibly mediated by multiple pathways because the pretreatment with these inhibitors decreased the endocytosis efficiency. The suppressions of endocytosis by amiloride hydrogen chloride and CytD were stronger, which suggested macropinocytosis and cytoskeleton played more important roles in the endocytosis of TPP@NPs. Notably, c(RGDfK) was also able to decrease the endocytosis efficiency by competing with ERGDS sequences of TPP@NPs. This phenomenon was an evidence to the integrin-mediated endocytosis pathway.

Apart from flow cytometry, the cellular uptake and distribution of TPP@NPs were visualized by 2D and 3D confocal laser scanning microscope (CLSM). After incubating A549 cells with free TPP or TPP@NPs, the cells were stained by Lyso-tracker Green and DAPI. From 2D CLSM images, red fluorescence was observed in the cytoplasm of cells treated with TPP@NPs (Fig. 4c) in contrast with nearly no detection of red fluorescence for free TPP-treated cells (Supplementary Fig. 17), indicating that the NPs not only enhanced cell uptake but also aid the dispersion of TPP. Multicellular tumor spheroid, a three-dimensional (3D) tumor model, was utilized to evaluate the penetration and internalization of TPP@NPs, which played significant roles in their therapeutic performances. 3D CLSM images not only proved that TPP@NPs locate in the cytoplasm of A549 cells but also indicated that TPP@NPs were able to penetrate the tumor and be uptaken by the internal cells in the tumor. As shown in Supplementary Fig. 18, red fluorescence perfused throughout the tumor spheroid, demonstrating that TPP@NPs possessed excellent tumor penetration, favorable for their in vivo PDT.

### In vitro PDT

Before we carried out in vitro PDT, the generation of ^1^O_2_ inside the cells by TPP@NPs was estimated by 2,7-dichlorofluorescein diacetate (DCFH-DA), which was oxidized to green-fluorescent dichlorofluorescein (DCF) in the presence of ^1^O_2_^44^. A549 cells were incubated with TPP@NPs and DCFH-DA, and these cells were subsequently exposed to 660 nm laser light (300 mW·cm^‒2^) for 120 s. Obvious green fluorescence was observed throughout the cells, a convincing evidence for the generation of ^1^O_2_. For comparison, negligible fluorescence was detected in the cells incubated with free TPP under the same irradiation conditions, illustrating that TPP inside the NPs was more capable of generating ^1^O_2_ than free TPP (Supplementary Fig. 19).

**Fig. 5:**
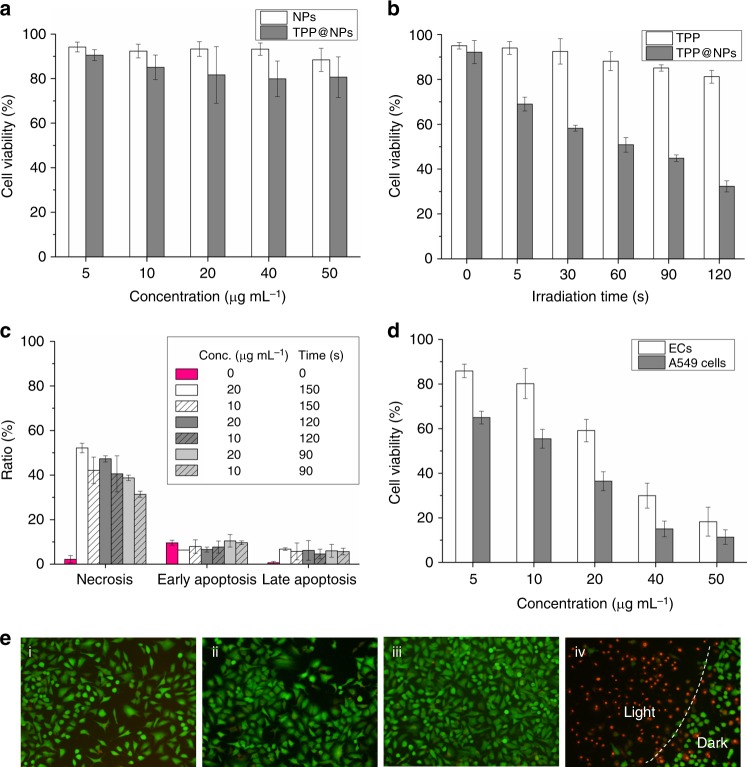
In vitro PDT. **a** Dark cytotoxicity of NPs and TPP@NPs to A549 cells. **b** Irradiation time-dependent light cytotoxicity of TPP and TPP@NPs to A549 cells. The concentration of TPP@NPs was 20.0 μg mL^‒1^. **c** Cell apoptosis analysis by flow cytometry of A549 cells treated with 20.0 μg mL^‒1^ of TPP@NPs and 120 s of irradiation. The cells were incubated for another 12 h after PDT. **d** Concentration-dependent light cytotoxicity of TPP@NPs to ECs and A549 cells to show target ability. The irradiation time was 120 s. **e** Living and dead cell imaging of A549 cells: (i) control, (ii) irradiation, (iii) TPP@NPs and (iv) TPP@NPs with irradiation. The boarder of laser was marked with white dash line in (iv) and the cells in the left side of the boarder were exposed to laser. The cell death occurred in the right side was ascribed to the flaw of laser collimation. Data are expressed as means ± s.d. (*n* = 3)

The PDT efficiency and cancer cell-target ability of the TPP@NPs were further investigated by 3-(4,5-dimethylthiazol-2-yl)-2,5-diphenyl tetrazolium bromide (MTT) assay. The dark cytotoxicities of both NPs and TPP@NPs against A549 cells were low, evidenced with the fact that over 80% of the cells survived when the concentration of the formulations reached 50 μg mL^‒1^, verifying their biocompatibility (Fig. 5a). Upon irradiation with 660 nm laser (300 mW cm^‒2^) for 120 s, limited cytotoxicity was monitored for free TPP, where the relative cell viability was as high as 80%. In sharp comparison, the cytotoxicity of TPP@NPs versus irradiation time significantly increased and nearly 70% of the cancer cells were killed, which indicated the PDT efficiency of TPP was remarkably enhanced by nanoformulation through a supramolecular strategy (Fig. 5b). To get better understanding of the PDT efficiency, cell apoptosis rate was measured by flow cytometry. A549 cells were incubated with TPP@NPs (10 and 20 μg mL^‒1^) for 6 h and then irradiated for different times (90, 120, and 150 s), respectively. After another 12 h incubation, these cells were labeled with Annexin V-FITC and propidium iodide (PI). As shown in Fig. 5c, PDT process induced significant necrosis/late apoptosis (>60%, 20 μg mL^‒1^ of TPP@NPs and 120 s irradiation), emphasizing that the light cytotoxicity against A549 cells was effective. In order to visualize the PDT effect, living/dead cell imaging was also carried out (Fig. 5e). Fluorescein diacetate (FDA) and PI were ultilized to stain A549 cells immediately after PDT (20.0 μg mL^‒1^ of TPP@NPs and 120 s of irradiation). Compared with negative control groups, obvious cell death was displayed by treating A549 cells with TPP@NPs followed by laser irradiation, whereas either laser irradiation or TPP@NPs exhibited moderate cell death. All these results confirmed that TPP@NPs was an excellent photosensitizer for PDT.

ERGDS sequences on the surface of TPP@NPs probably showed cancer cell-targeting ability to integrin-overexpressed cancer cells. Therefore, we further carried out the concentration-dependent PDT process against A549 cells and endothelial cells (ECs, as normal cells) to elucidate the selectivity of TPP@NPs. According to the MTT data (Fig. 5d), concentration-dependent light cytotoxicity of TPP@NPs to ECs was lower than it to A549 cells that over 50% ECs survived at 20.0 μg mL^‒1^. Higher concentration caused higher cytotoxicity and lower selectivity, evidenced with the fact that 11% of A549 cells and 18% of ECs survived at 50.0 μg mL^‒1^. This could be ascribed to the promoted endocytosis under high concentration.

### In vivo PDT

**Fig. 6:**
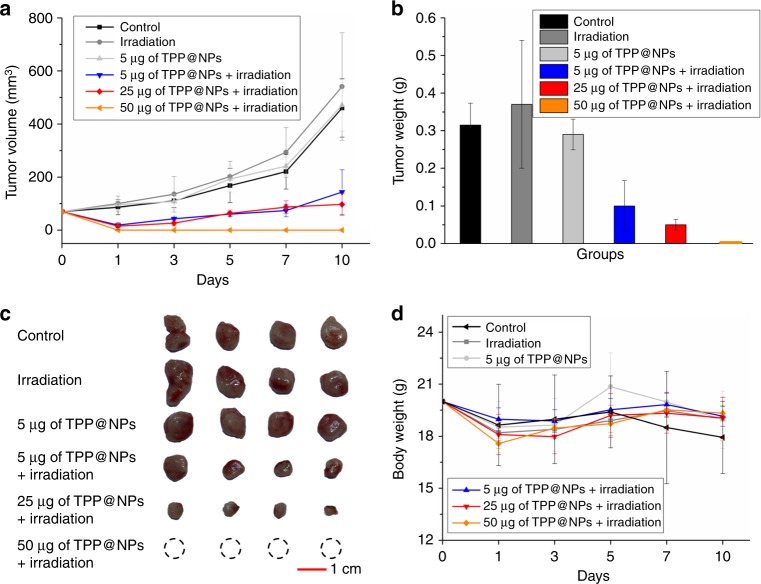
In vivo PDT results of 4T1 tumor-bearing mice. **a** Tumor volume changes and **b** final tumor weights of the mice bearing 4T1 tumors treated with different formulations and procedures after one injection. **c** Images of final tumors. The scale bar was shown in **c**. **d** Body weight changes of the mice bearing 4T1 tumors treated with different formulations and procedures after one injection. Data are expressed as means ± s.d. (*n* = 3)

In vivo anti-tumor efficiency of TPP@NPs was further evaluated using 4T1 tumor-bearing mice. 4T1 cells are very aggressive to living tissues, which makes them highly tumorigenic and can metastasize from the original tumor to multiple sites including blood, liver, lung, brain, and bone. After metastasis, the 5-year survival rate is lower than 20% because if only the primary breast tumor is removed, the multiple metastatic foci are still intact and fatal. Therefore, finding new efficient treatments for breast cancer is urgent and meaningful, and thus we chose invasive 4T1 animal model to exam the anti-tumor efficiency of the supramolecular peptide. Besides, integrin is also overexpressed in 4T1 cells, benefiting targeting ability and internalization of TPP@NPs through RGD sequence. The 4T1 tumor-bearing mice were divided into six groups randomly when the tumor volume reached ~100 mm^3^, and the mice were treated with (i) PBS (control), (ii) irradiation, (iii) 5 μg of TPP@NPs, (iv) 5 μg of TPP@NPs + irradiation, (v) 25 μg of TPP@NPs + irradiation, and (vi) 50 μg of TPP@NPs + irradiation, respectively. The second and the third groups were used to estimate the effect of TPP@NPs or irradiation, and the last three groups were designed to study the dosage-dependent anti-tumor efficiency. The tumor was irradiated with laser (660 nm, 300 mW cm^‒2^) for 6 min. As shown in Fig. 6a, the average tumor volumes of the mice treated with PBS, 5 μg of TPP@NPs or laser increased rapidly, indicating either 5 μg of TPP@NPs or irradiation didn’t influence the tumor growth. In the sharp contrast, the injection of TPP@NPs followed by irradiation decreased the tumor volumes with a dosage-dependent efficiency: 5 μg of TPP@NPs with irradiation was able to decrease the tumor volume but the tumor recurrence occurred quickly, whereas the delay of the tumor growth and recurrence was exhibited by increasing the injection dosage to 25 μg. Moreover, when 50 μg of TPP@NPs was dosed, the tumor was completely eliminated after 1 day post-PDT, and the tumor recurrence didn’t occur during the therapy period. According to the final tumor weight, compared with the control group, about 73, 86, and 100% of 4T1 tumor reduction was achieved by the dosage of 5, 25, and 50 μg of TPP@NPs with irradiation, and yet irradiation or 5 μg of TPP@NPs showed negligible anti-tumor effect. The reasons for high anti-tumor efficiency could be attributed to the synergic effects including enhanced endocytosis by ERGDS sequence, promoted photoactivity of TPP and decreased GSH level by disulfide bonds of TPP@NPs. The biocompatibility of TPP@NPs was illustrated by the body weight loss of mice after treatment with TPP@NPs and irradiation (Fig. 6d). Compared with the mice treated with PBS (loss 10% weight after 10 days), the mice treated with PDT regained body weight to normal level during the therapy period, indicating the excellent biocompatibility and low systemic toxicity of TPP@NPs due to the rational design of the therapeutic system and fast degradation of peptides.

In summary, the self-assembly morphologies of this supramolecular peptide including sheets and NPs could be conveniently controlled by adding P5 and heating rather than tedious covalent modification of peptides. Moreover, the cysteine units on the PyP were oxidized to disulfide cross-linked shells that prohibited the collapse of the NPs after the temperature decrease. The disulfide bonds also showed responsiveness to GSH that potentially consumed intracellular GSH to synergically enhance PDT efficiency. To extend the application, we loaded TPP into the NPs. The hydrophobic core of the NPs compensated the π–π stacking between TPP and recovered the capability to generate ^1^O_2_. Compared with free TPP, TPP@NPs showed enhanced internalization and PDT efficiency to A549 cells. The cancer cell-target ability was also tested that the light cytotoxicity of TPP@NPs against A549 cells was higher than it against normal cells. In vivo PDT revealed high anti-tumor efficiency against 4T1 tumor and biocompatibility. Therefore, we exhibited a promising method to fabricate supramolecular peptides with controllable self-assembly behavior and PDT functionality. It’s convincing that pillararene-based host−guest chemistry has more potential in controlling peptide self-assembly and bio-applications.

This supramolecular peptide Lego also provides a blueprint to construct peptide-based nanotheranostics. By changing the Lego blocks, various kinds of functional building blocks can be introduced into the supramolecular peptide via precise molecular recognition. For examples, by functionalizing pillararenes, imaging probe, radioactive agent, or anti-tumor prodrug can be incorporated into the supramolecular peptide without covalent synthesis and purification. The delivery, pharmacokinetic behaviors, biodistributions, excretion, and therapeutic results of the nanotheranostic systems can be monitored with the assistance of molecular imaging. In addition, other therapeutic modalities including chemotherapy, gene therapy, phototheramotherapy, and immunotherapy are able to be combined into the same platform to realize synergistic efficiency. Besides, the function of the peptide is completely maintained that either self-assembly or biological behavior is as normal as designed, which is also an advantage of the molecular Lego-constructed supramolecular peptide. Moreover, compared with covalent prepared polymeric nanomedicine, the metabolism and elimination of peptide-based nanostructures are easier and faster, greatly avoiding the immunotoxicity and side effect. Therefore, the Lego block bridge the gap between biology and supramolecular chemistry and offers a simple approach to broad the application of peptides and pillararene-based biomaterials.

## Method

### Control on peptide self-assembly

PA or PyP was dissolved in PBS (*c* = 10 mM) to form transparent solutions. After adding 2.0 molar equivalent of CaCl_2_, the solution was sonicated and stored for 30 min. One molar equivalent of P5 was added to the solution of PyP (*c* = 10 mM, 1 mL) and followed by heating in an air-flow oven for one hour at 45 °C. SEM or TEM investigation was carried out by dropping the solutions on silica wafers or copper matrices and frozen dried.

### TPP loading

A reprecipitation method was used in TPP loading. One milligram of TPP in 0.3 mL of acetone was added into an aqueous solution (0.6 mL) of PyP/P5 (10 mg). The mixture was subsequently added to PBS (10 mL) at 45 °C under oxygen atmosphere and shaken for 12 h. Excess acetone and TPP were dialyzed against PBS. To measure the loading efficiency, TPP@NPs (*ca*. 10 mg) were frozen dried and re-dissolved in 100 mL of acetone to dissolve encapsulated TPP. The concentration of TPP was determined by UV-vis spectrometry according to the standard curve of TPP in acetone. The loading efficiency, calculated by (loaded TPP/ total TPP) × 100%, was determined to be 65%. The data are shown in Section S7.

### PDT procedure, cell apoptosis analysis, and living and dead cell imaging

A549 cells and ECs were purchased from American Type Culture Collection (ATCC). After seeding the cells at a density of 10^4^ cells per well in a 96-well plate and incubated for 24 h, TPP@NPs were added at various concentrations from 5.0 to 50 μg mL^‒1^ and incubated for 6 h at 37 ^o^C in dark. Next, the cells were washed with PBS and added with culture medium (phenol red free), and the cells were irradiated with a 660 nm laser light (300 mW·cm^‒2^) for 120 s. After the cells were incubated for another 12 h, the cell viability was determined by MTT assay. For irradiation time-dependent cytotoxicity, 20 μg mL^‒1^ of TPP or TPP@NPs were added and the cells were irradiated for various times from 5 s to 120 s. For cell apoptosis analysis, PDT procedure was as the same as above. The cells were incubated for 12 h after PDT and then stained by Annexin V-FITC/PI. The apoptosis rate was analyzed by flow cytometry. For living and dead cell imaging, the same PDT procedure was carried out but the cells were immediately stained by FDA/PI after irradiation. The stained cells were imaged under fluorescence microscope.

### Other methods

Other information about syntheses, characterisations, in vitro studies, and in vivo investigations are given in Supplementary Information. Animal care and handing procedures were in agreement with the guidelines evaluated and approved by the ethics committee of Zhejiang University. Study protocols involving animals were approved by the Zhejiang University Animal Care and Use Committee. No third party material is included.

### Reporting summary

Further information on research design is available in the Nature Research Reporting Summary linked to this article.

## Supplementary information

Supplementary information

Reporting Summary

## Data Availability

All data are available from the authors upon reasonable request.
