# Supplementary material for: Supramolecular peptide constructed by molecular Lego allowing programmable self-assembly for photodynamic therapy
Source: Nat Commun. 2019 Jun 3;10:2412. doi: 10.1038/s41467-019-10385-9 (PMC6546686; doi:10.1038/s41467-019-10385-9)
Supplement: Supplementary file 1 — Supplementary information [file 41467_2019_10385_MOESM1_ESM.pdf]

Supramolecular peptide constructed by molecular Lego allowing programmable self-assembly for photodynamic therapy

Feihe Huang, *et al*.

State Key Laboratory of Chemical Engineering, Center for Chemistry of High-Performance & Novel Materials, Department of Chemistry, Zhejiang University, Hangzhou 310027, P. R. China. E-mail: fhuang@zju.edu.cn.

MOE Key Laboratory of Macromolecular Synthesis and Functionalization, Department of Polymer Science and Engineering, Zhejiang University, Hangzhou 310027, P. R. China. E-mail: zwmao@zju.edu.cn

Laboratory of Molecular Imaging and Nanomedicine, National Institute of Biomedical Imaging and Bioengineering, National Institutes of Health, Bethesda, Maryland 20892, United States. E-mail: guocan.yu@nih.gov

Supplementary (17 pages)

1.	*Materials and methods*	S3
2.	*Synthesis of* ***PyP****,* ***P5*** *and* ***G1***	S6
3.	*NOESY NMR spectrum of* ***P5⊃G1***	S10
4.	*Isothermal titration calorimetry (ITC) experiment on* ***P5⊃G1***	S10
5.	*Temperature-dependent* ^{1}H *NMR spectra and transmittance of* ***P5⊃G1***	S11
6.	*Dynamic light scattering (DLS) analysis of nanoparticles (NPs)*	S11
7.	*Reduction experiment of NPs*	S12
8.	*Loading efficiency of TPP in NPs*	S12
9.	*UV-vis spectra of free TPP and TPP@NPs*	S13
10.	*Fluorescence spectra of free TPP and TPP@NPs*	S13
11.	*Detection of* $^{1}O_2$ *generation by 1,3-diphenylisobenzofuran (DPBF)*	S14
12.	*2D CLSM images of TPP in A549 cells*	S14
13.	*3D CLSM images of TPP@NPs in the A549 tumor spheroid*	S15
14.	*Intracellular* $^{1}O_2$ *detection by 2,7-dichlorofluorescein diacetate (DCFH-DA)*	S16
15.	*Flow cytometry analysis of endocytosis and apoptosis rate*	S16
16.	*References*	S17

1. Materials and methods

All reagents were commercially available and used as supplied without further purification. Peptide Br-$C_{10}H_{21}$CO-*GGGGGGGCCERGDS* (**PA**) was prepared by solid phase peptide synthesis, purified by HPLC and confirmed by LCMS by Sangon Biotech Co., Ltd (Shanghai, China). ^{1}H NMR and ^{13}C HMR spectra were recorded with a Bruker Avance DMX 400 spectrometer using the deuterated solvent as the lock and the residual solvent or TMS as the internal reference. Transmission electron microscopic (TEM) investigations were carried out on a HITACHI HT-7700 instrument. Scanning electron microscopy (SEM) investigations were carried out on a JEOL 6390LV instrument. UV-vis spectra were taken on a PerkinElmer Lambda 35 UV-vis spectrophotometer. The fluorescence experiments were conducted on a RF-5301 spectrofluorophotometer (Shimadzu Corporation, Japan). The ITC experiment was performed on a VP-ITC micro-calorimeter (Microcal, USA). Photographs were taken with a Cannon 550D. Dynamic light scattering was carried out on a Malvern Nanosizer S instrument at room temperature. Fluorescence images were taken with Olympus IX71 inverted fluorescence microscope. 2D and 3D CLSM images were taken on a Zeiss CLSM system. Flow cytometry was carried out on Cytoflex S (Berkman, Germany). Cells were purchased from ATCC and authenticated by STR. No mycoplasma contamination. The pH of PBS in all experiment was 7.4. The 660 nm laser density in all irradiation procedures was 300 mW cm^{-2}.

Cell culture, flow cytometry and confocal laser scanning microscopy. A549 cells were cultured by Dulbecco's modified Eagle's medium/HamF12 (DMEM/F12) containing 10% fetal bovine serum (FBS), 100 μg mL^{-1} of penicilin and 100 U of streptomycin at 37 ^{o}C under a humidified atmosphere of 95% air : 5% CO_2. To estimate the endocytosis of TPP@NPs, A549 cells were seeded in a 24-well plate at a density of 6×10^4 cells per well and incubated. Then the cells were treated with TPP or TPP@NPs and incubated for another 1 to 6 h. After that, the cells were analyzed by flow cytometry. To study the endocytosis pathways of TPP@NPs, different endocytosis inhibitors

(concentration of inhibitors: amantadine HCl, 10^{-3} mM; genistein, 100 mM; amiloride HCl, 2 mM, CytD, 10 μg mL^{-1}; c(RGDfK), 50 nM) were used to pretreat A549 cells for 45 min before incubation with TPP@NPs. The distribution of TPP@NPs inside the cells was visualized by 2D and 3D CLSM. For 2D CLSM, A549 cells were seeded in a 6-well plate at a density of 10^4 cells per well and incubated with TPP@NPs for 6 h. After washing with PBS, the cells were stained with Lyso-tracker green, fixed with 4% formaldehyde and then stained with DAPI. For 3D CLSM, the A549 tumor spheroid was cultured on sphera 96-well U-bottom 3D cell culture plate for 4 days and then stained by TPP@NPs for 12 h, Lyso-tracker Green for 2 h and Hoechst 33342 for 1 h.

Target ability analysis of TPP@NPs. We used endothelial cells (ECs) as normal cells to study the concentration-dependent light cytotoxicity. The cell culture and PDT process were as the same as them for A549 cells. The cell viability of ECs was estimated by MTT assay.

In vivo PDT. 4T1 tumor-bearing mice (24 mice, initial average tumor volume: 70 mm^3; average body weight: 20.0 g) were divided into six groups randomly: (i) control, (ii) irradiation only, (iii) 5 μg of TPP@NPs, (iv) 5 μg of TPP@NPs + irradiation, (v) 25 μg of TPP@NPs + irradiation and (vi) 50 μg of TPP@NPs + irradiation. Group (i) and (ii) were injected with 50 μL of PBS. Group (iii) to (vi) were injected with 50 μL of TPP@NPs with concentrations of 0.1, 0.5 and 1.0 mg mL^{-1}, respectively. The injection was intratumoral injection. After 30 min, the mice were irradiated under 660 nm laser light at 300 mW cm^{-2} for 3 min, and another 3 min of irradiation after an interval of 1 min (for avoiding temperature rising under laser). The mice were normally fed after PDT and the body weight and tumor volume were recorded at determined time. After 10 days, the mice were sacrificed and tumors were collected to image and measure the size and weight.

We would like to use *in vivo* PDT to estimate the anti-tumor efficiency of the supramolecular peptide and to illustrate this therapeutic system definitely having more

potentials in cancer treatment. Considering intratumoral injection is more effective, we chose intratumoral injection to dose TPP@NPs.

2. Synthesis of PyP, P5 and G1

Preparation of 4-methylpyridinium-$C_{10}H_{21}$CO-*GGGGGGGCCERGDS* bromide (**PyP**): A solution of **PA** (50 mg) in 200 μL of DMF was added 50 μL of 4-methylpyridine and stirred at 60 ℃ under argon atmosphere overnight. DMF was subsequently removed under high vacuum and 200 μL of water was added to the residual solid to dissolve the product. Unreacted **PA** didn't dissolve in water at neutral condition. After the solvent was removed in vacuum, near pure **PyP** was obtained as a pale yellow solid (31 mg, yield 58 %). ^{1}H NMR (600 MHz, D_2O, 298 K) δ(ppm): 8.49 (d, J = 6 Hz, 2H), 7.73 (d, J = 6 Hz, 2H), 4.72 (m, 1H), 4.39 (m, 3H), 4.25 (m, 1H), 4.20 (m, 1H), 3.85 (dd, J = 5 Hz, J = 5 Hz, 15H), 3.78 (m, 1H), 3.09 (t, J = 7 Hz, 2H), 2.83 (d, J = 5 Hz, 2H), 2.77 (d, J = 8 Hz, 2H), 2.51 (s, 3H), 2.36 (m, 2H), 2.19 (m, 2H), 2.11 – 1.95 (m, 2H), 1.85 (m, 6H), 1.58 – 1.50 (m, 2H), 1.45 (m, 2H), 1.15 (m, 12H).

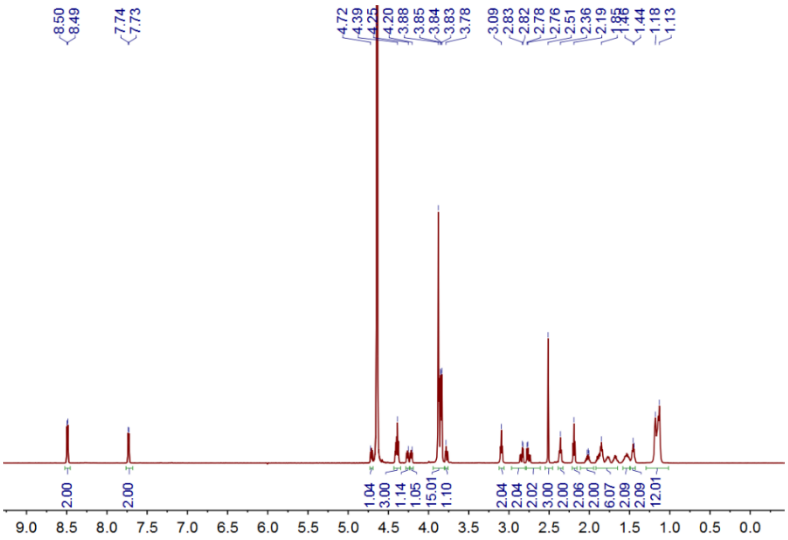

Supplementary Figure 1. ^{1}H NMR spectrum (400 MHz, D_2O, 298K) of **PyP**.

The mass spectra of **PA** and **PyP** were shown below:

LCMS of **PA** ($C_{51}H_{84}N_{17}O_{21}S_2Br$): *m/z* calcd for [M + H]+ $C_{51}H_{85}N_{17}O_{21}S_2Br^+$, 1415.93, found 1415.50.

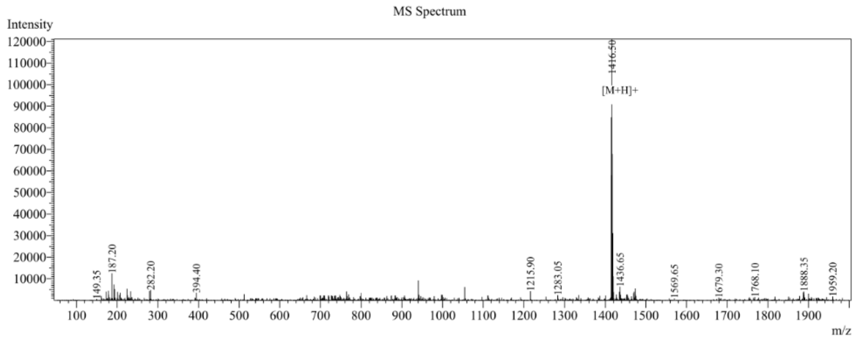

Supplementary Figure 2. LCMS spectrum of **PA**.

LCMS of **PyP** ($C_{57}H_{91}N_{18}O_{21}S_2Br$): *m/z* calcd for $[M – Br + 2H]^{2+}$ $C_{57}H_{92}N_{18}O_{21}S_2^{2+}$, 714.30, found 714.70.

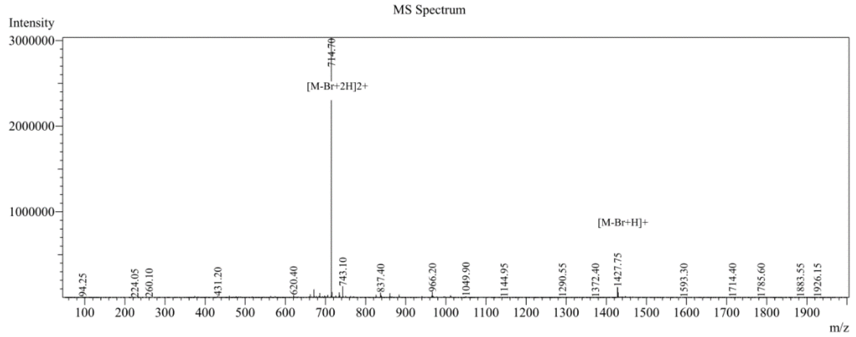

Supplementary Figure 3. LCMS spectrum of **PyP**.

Preparation of *per*-tri(ethyleneoxide)pillar[5]arene (**P5**): P5 was synthesized according to a published procedure.[S1] Briefly, *per*-hydroxylpillar[5]arene was stirred with excess 2-(2’-(2’’-methoxyethoxy)ethoxy)ethyl 4-methylbenzenesulfonate[S2] in presence of K_2CO_3 in CH_3CN under reflux for 4 days. Crude product was purified by column chromatography on silica gel using dichloromethane: methanol = 30: 1 as the eluent to obtain **P5** as light yellow oil in a moderate yield. MALDI-TOF MS of **P5** ($C_{105}H_{170}O_{40}$): *m/z* calcd for $[M – e]^+$ $C_{105}H_{170}O_{40}^+$, 2072.13, found 2071.87; *m/z* calcd for $[M + Na]^+$ $C_{105}H_{170}O_{40}Na^+$, 2095.12, found 2094.87.

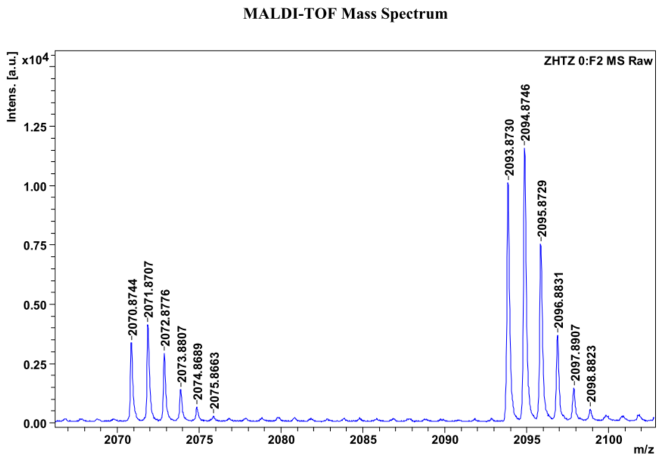

Supplementary Figure 4. MALDI-TOF mass spectrum of **P5** (left peaks: $[M - e]^+$; right peaks: $[M + Na]^+$).

Preparation of 4-methyl-1-propylpyridinium bromide (**G1**): 1-Bromopropane (1.2 g, 10 mmol) was added to a solution of 4-methylpyridine (0.46 g, 5 mmol) in 20 mL of CH_3CN. The mixture was refluxed overnight. After cooling to room temperature, the solvent was removed by an evaporator and the oily product (1.0 g, 93 %) was obtained by extraction with water against dichloromethane. ^{1}H NMR (400 MHz, D_2O, 298K) δ (ppm): 8.48 (d, J = 6 Hz, 2H), 7.72 (d, J = 6 Hz, 2H), 4.35 (t, J = 7 Hz, 2H), 2.50 (s, 3H), 1.86 (d, J = 7 Hz, 2H), 0.79 (t, J = 7 Hz, 3H). ^{13}C NMR (101 MHz, D_2O, 298K) δ (ppm): 159.85, 143.06, 128.58, 62.39, 24.03, 21.29, 9.68. HRESIMS: m/z calcd for $[M - Br]^+$ $C_{19}H_{32}N_3O_2^+$, 136.1126, found 136.1117, error –7 ppm.

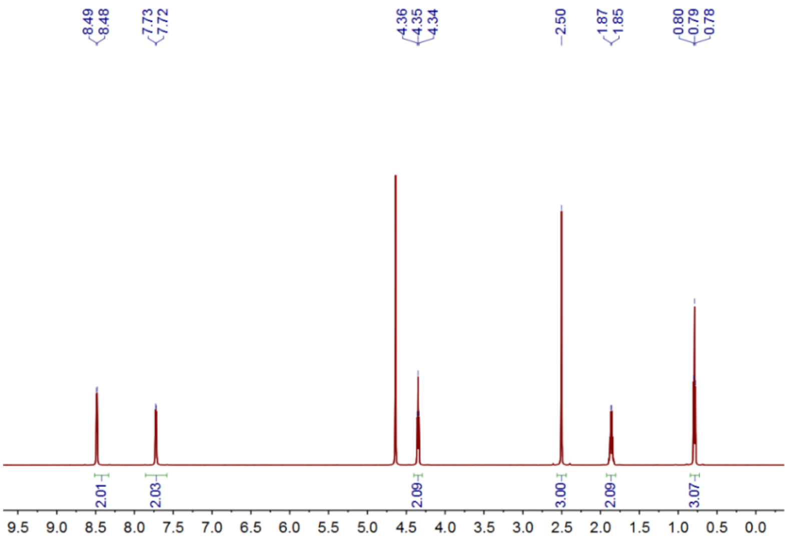

Supplementary Figure 5. ^{1}H NMR spectrum (400 MHz, D_2O, 298K) of **G1**.

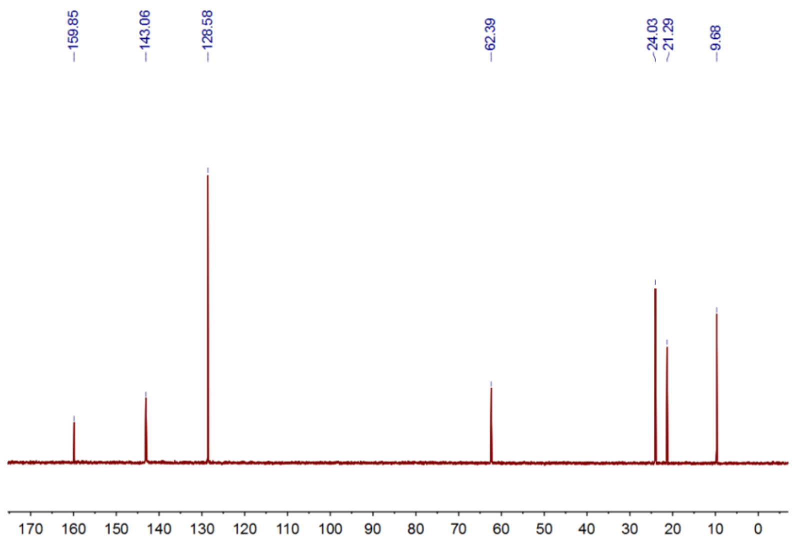

Supplementary Figure 6. ^{13}C NMR spectrum (101 MHz, D_2O, 298K) of **G1**.

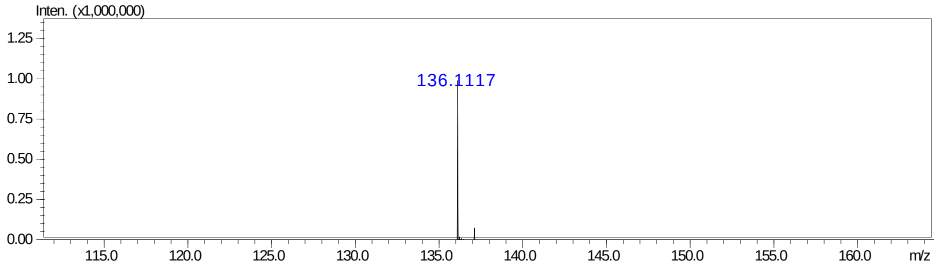

Supplementary Figure 7. HRESIMS spectrum of **G1**.

3. NOESY NMR spectrum of P5⊃G1

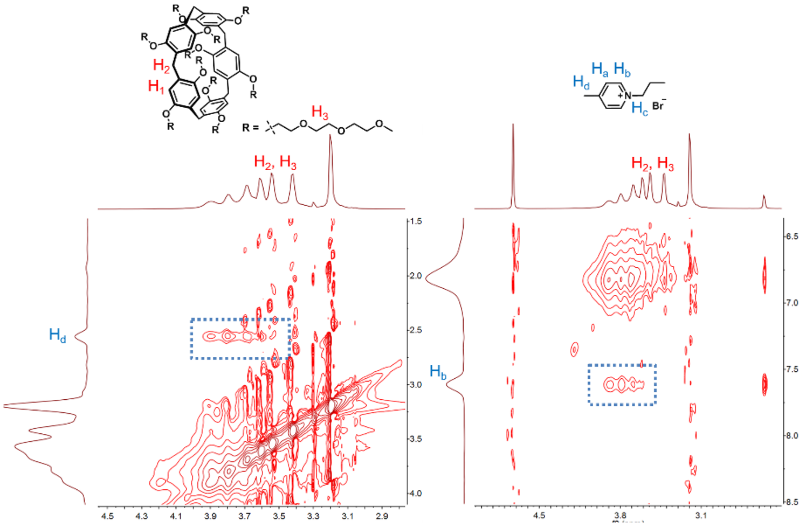

Supplementary Figure 8. Partially enlarged NOESY NMR spectrum (400 MHz, D_2O, 298K) of **P5⊃G1**. The NOE correlation signals are marked in the spectrum.

4. Isothermal titration calorimetry (ITC) experiment on P5⊃G1

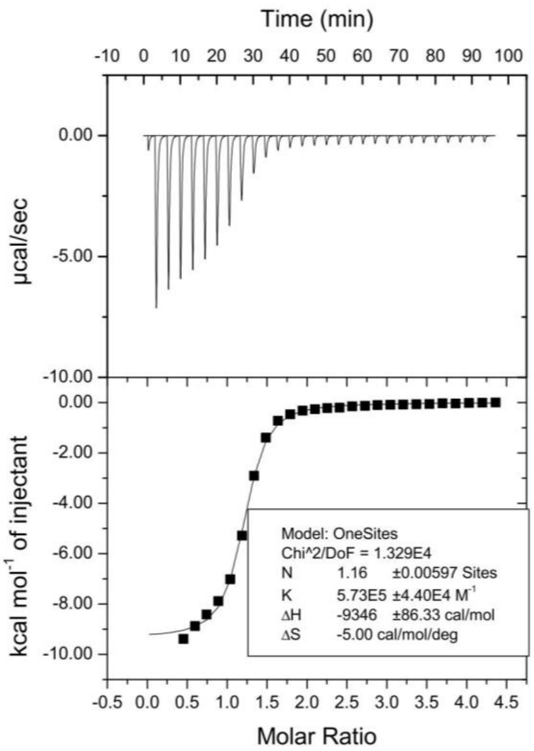

Supplementary Figure 9. ITC experiment of **P5⊃G1**. From this experiment, the complexation stoichiometry between **P5** and **G1** was determined to be 1:1, and the association constant (K_a) was 5.73×10^5 M^{-1}.

5. Temperature-dependent ^{1}H NMR spectra and transmittance of P5⊃G1

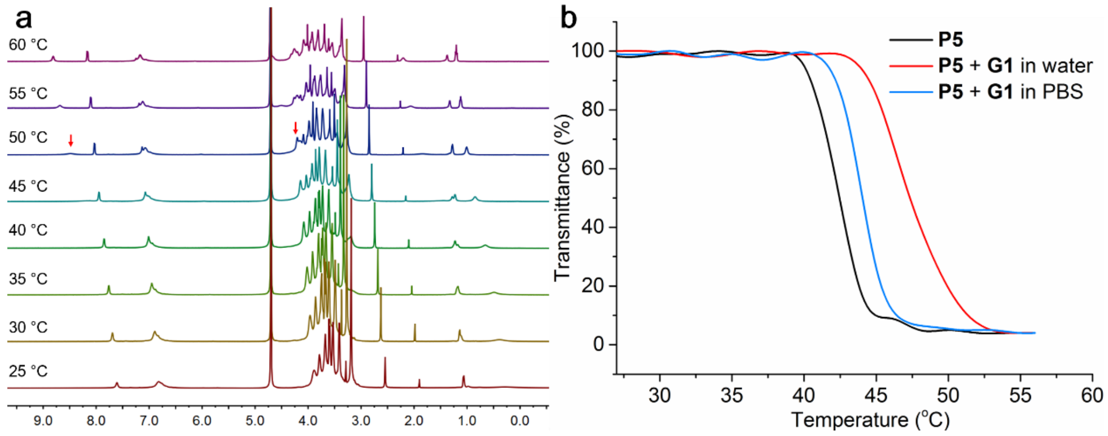

Supplementary Figure 10. **a** Temperature-dependent ^{1}H NMR spectra (400 MHz, D_2O) of **P5⊃G1**. The temperature values are stated and the peaks related to de-threading **G1** are marked with red arrows in the spectra. **b** Transmittance at 700 nm upon heating the solutions of **P5** (black line), **P5** + **G1** (in water, red line) and **P5** + **G1** (in PBS, blue line).

6. Dynamic light scattering (DLS) analysis of nanoparticles (NPs)

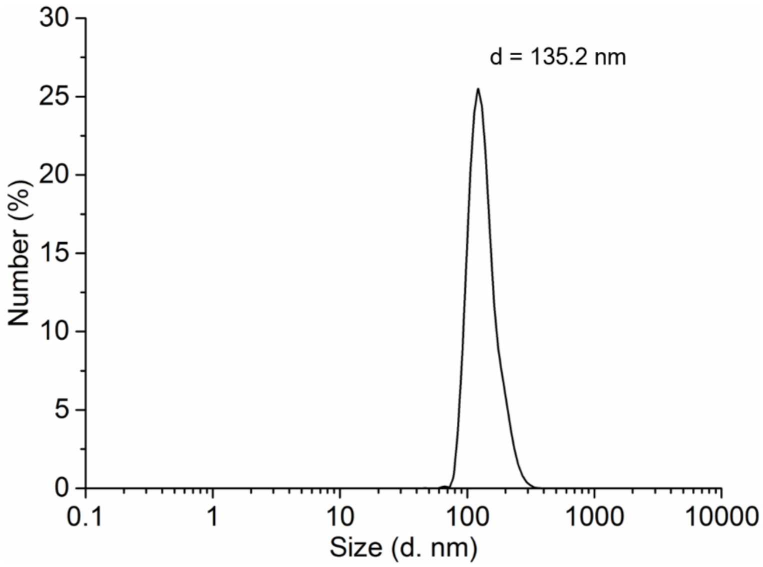

Supplementary Figure 11. DLS analysis of NPs. The average size is 135 nm.

7. Reduction experiment of NPs

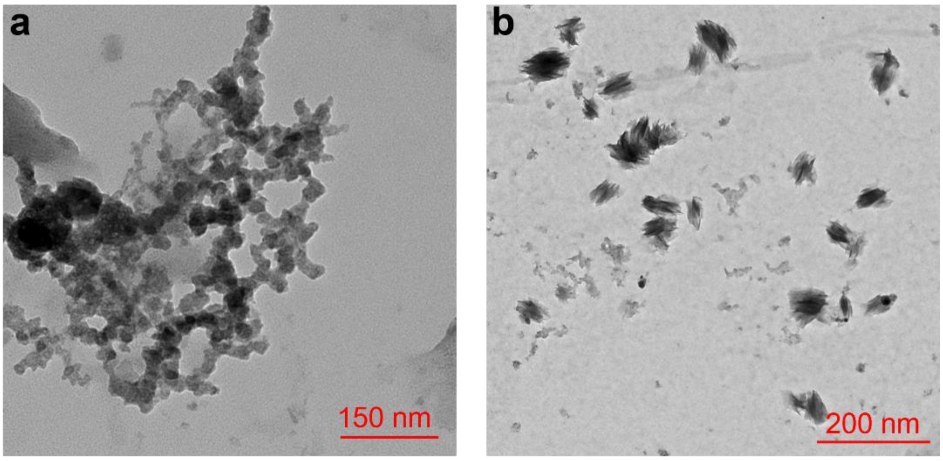

Supplementary Figure 12. TEM images of NPs after adding 3.0 equal molar of **a** GSH and **b** TCEP.

8. Loading efficiency of TPP in NPs

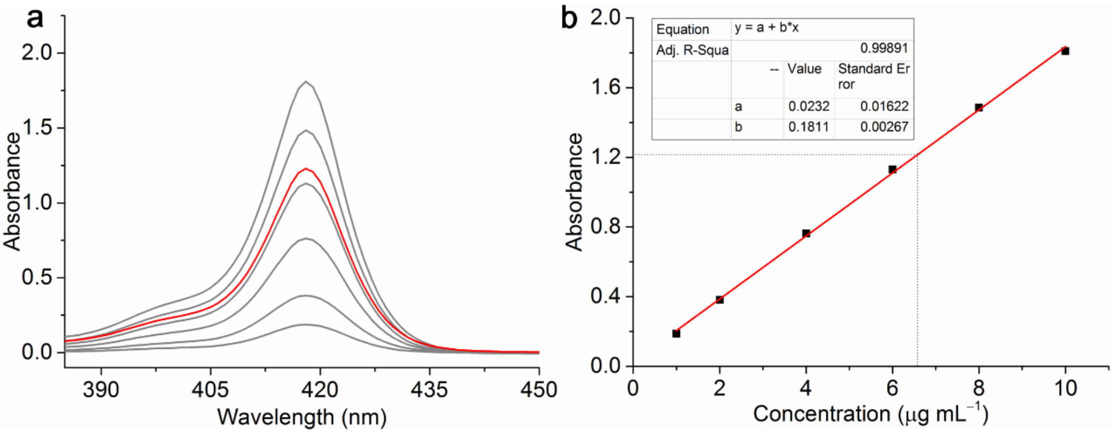

Supplementary Figure 13. a UV absorbance of TPP for the standard curve (gray lines) and the total TPP in 10 mg of TPP@NPs in 100 mL of acetone (red line). **b** The standard curve of UV absorbance of TPP in acetone versus concentrations. The weight of TPP in 10 mg of TPP@NPs was determined to be 0.65 mg.

9. UV-vis spectra of free TPP and TPP@NPs

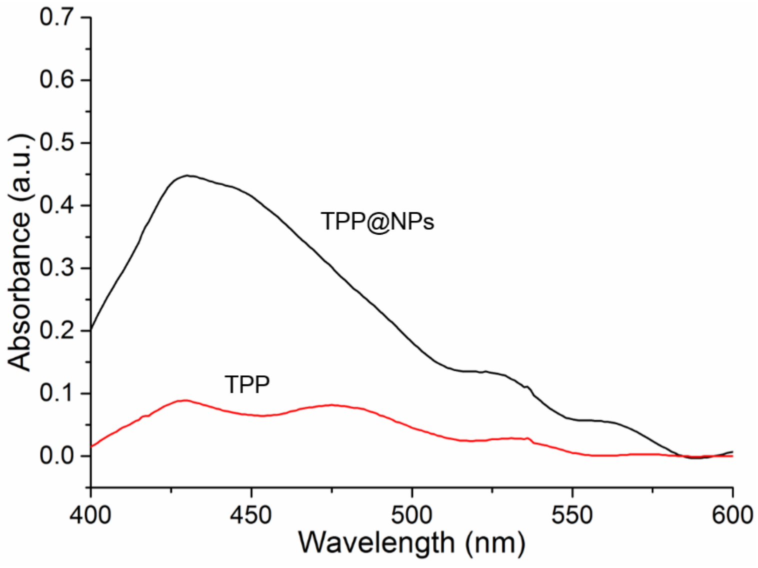

Supplementary Figure 14. UV-vis spectra of free TPP (saturated solution in PBS, black line) and TPP@NPs (1.0 mg mL^{-1} in PBS, red line).

10. Fluorescence spectra of free TPP and TPP@NPs

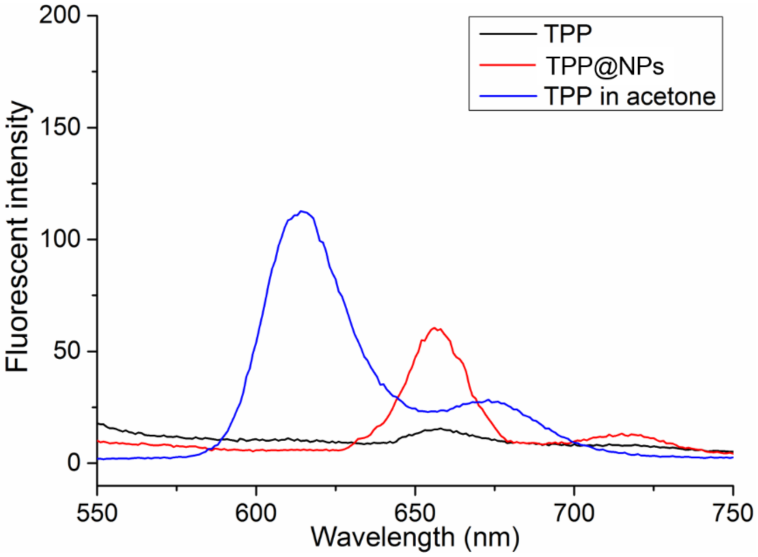

Supplementary Figure 15. Fluorescence spectra of free TPP (saturated solution in PBS, black line), TPP@NPs (1.0 mg mL^{-1}, red line) in PBS and free TPP (0.06 mg mL^{-1}, blue line) in acetone.

11. Detection of $^1O_2$ generation by 1,3-diphenylisobenzofuran (DPBF)

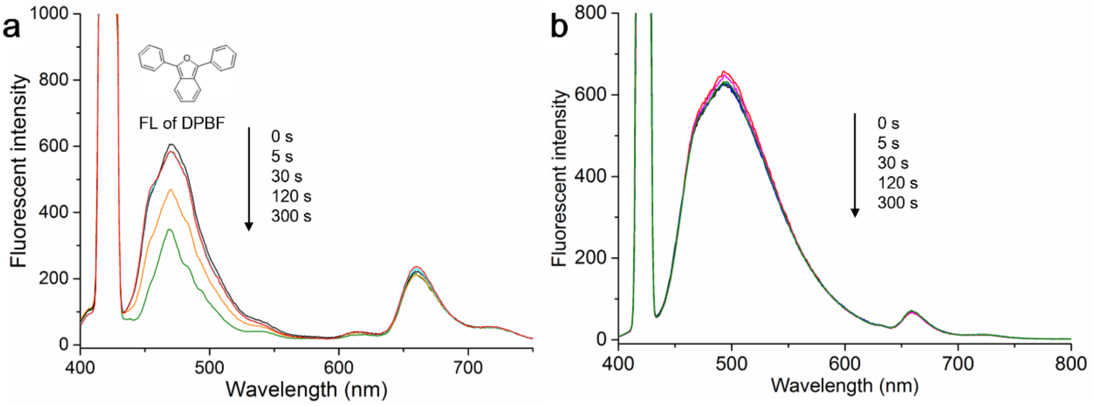

Supplementary Figure 16. Fluorescence spectra of DPBF in presence of **a** TPP@NPs and **b** free TPP under irradiation with 660 nm laser light in PBS (containing 1 % DMF to dissolve DPBF). During the irradiation process, the fluorescence intensity at 470 nm in **a** decreased, indicating the oxidation degradation of DPBF and the generation of $^1O_2$ by TPP@NPs.

12. 2D CLSM images of TPP in A549 cells

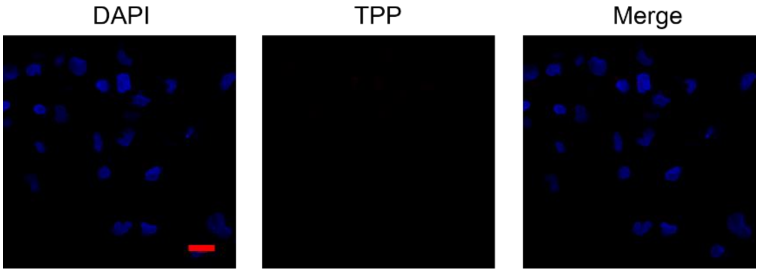

Supplementary Figure 17. CLSM images of A549 cells incubated with TPP and then fixed with 4 % of formaldehyde and stained with DAPI. No red fluorescence was observed, illustrating weak internalization of TPP to A549 cells.

13. 3D CLSM images of TPP@NPs in an A549 tumor spheroid

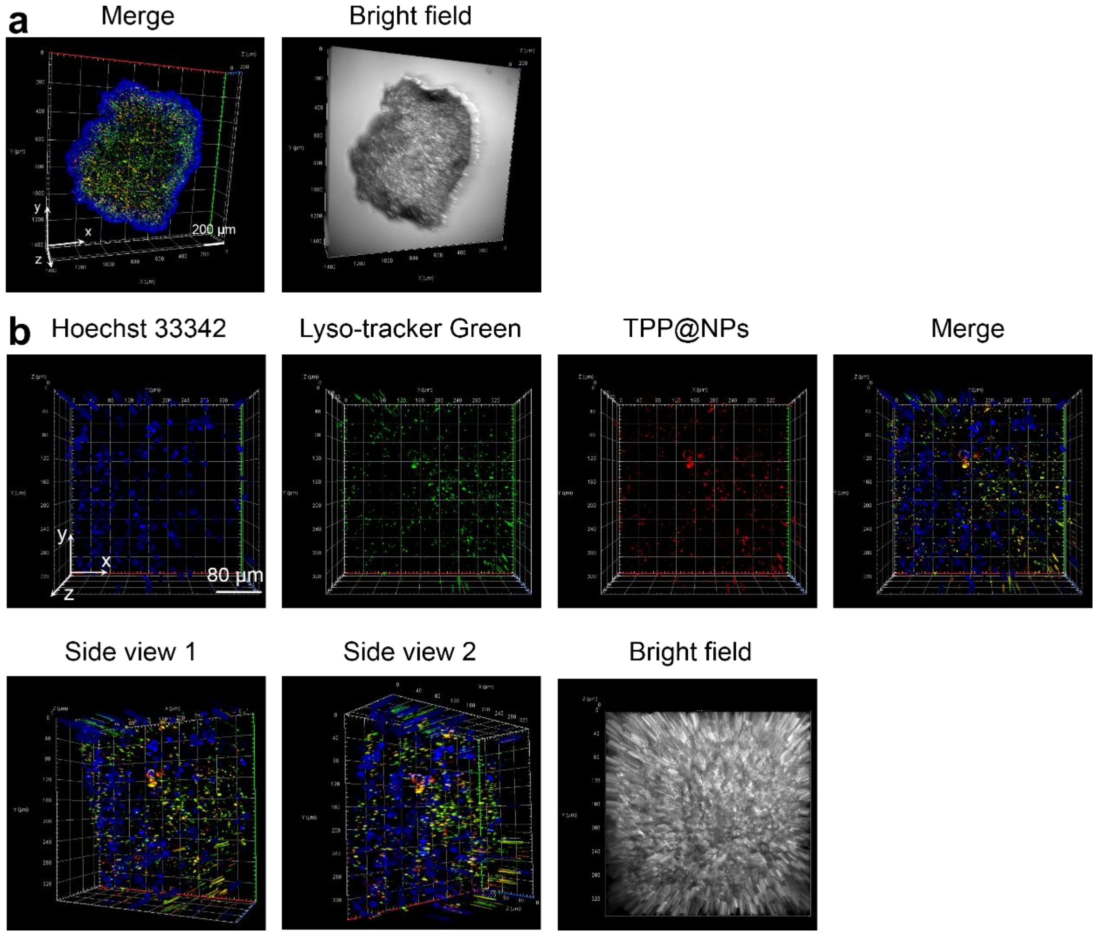

Supplementary Figure 18. 3D CLSM images of an A549 tumor spheroid stained with TPP@NPs (red), Lyso-tracker Green (staining lysosomes, green) and Hoechst 33342 (staining nucleus, blue). **a** The tumor spheroid used for 3D CLSM imaging. **b** Enlarged images of the tumor spheroid. The images prove that TPP@NPs locate in the cytoplasm of A549 cells. The fluorescence of TPP@NPs from the internal cells of the tumor spheroid indicate that TPP@NPs can be uptaken by the internal cells in the A549 tumor (deep penetration). The x, y and z axis and scale bar are shown in the images.

14. Intracellular $^1O_2$ detection by 2,7-dichlorofluorescein diacetate (DCFH-DA)

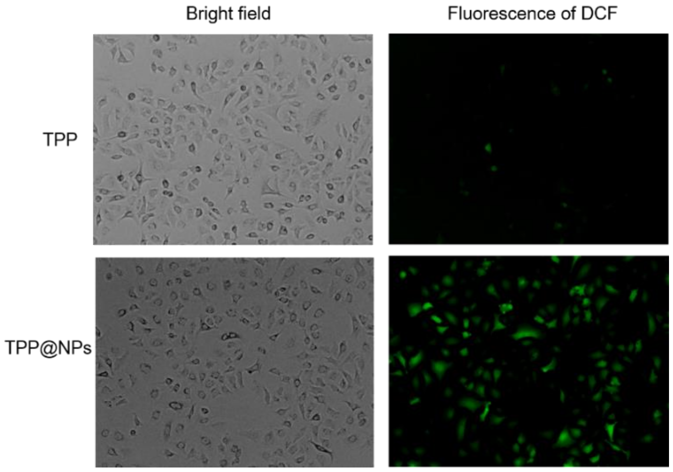

Supplementary Figure 19. Fluorescence microscope images of A549 cells incubated with TPP or TPP@NPs after irradiation with 660 nm laser light for 120 s. The green fluorescence indicates DCFH-DA is oxidized to DCF in the presence of TPP@NPs.

15. Flow cytometry analysis of endocytosis and apoptosis rate

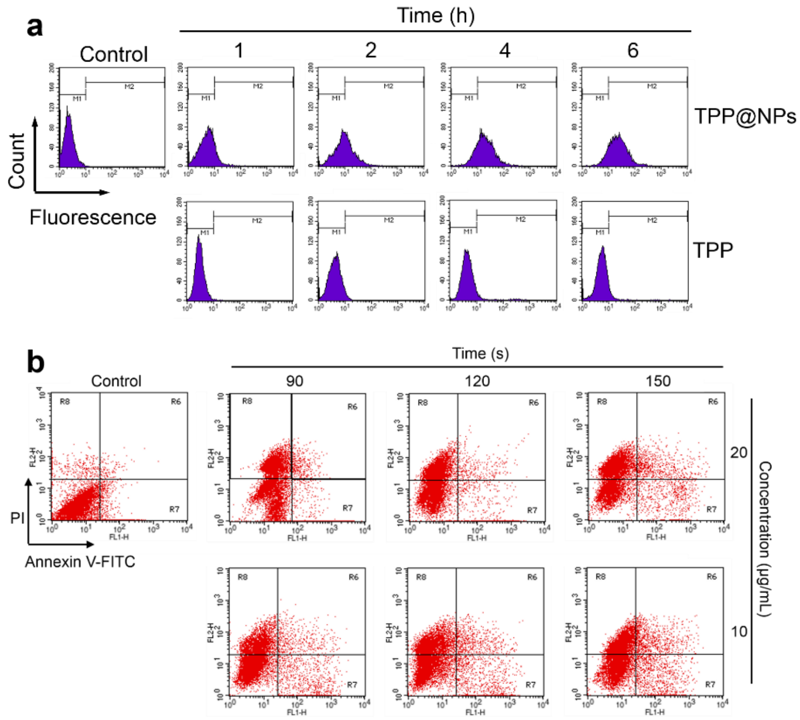

Supplementary Figure 20. Flow cytometry of A549 cells in the experiments of **a** time-dependent endocytosis of TPP and TPP@NPs and **b** apoptosis rate measurement for PDT efficiency.

16. References

S1. Ogoshi, T., Kida, K. & Yamagishi, T. Photoreversible Switching of the Lower Critical Solution Temperature in a Photoresponsive Host–Guest System of Pillar[6]arene with Triethylene Oxide Substituents and an Azobenzene Derivative. *J. Am. Chem. Soc.* **134**, 20146–20150 (2012).

S2. Kohmoto, S., Mori, E. & Kishikawa, K. Room-temperature discotic nematic liquid crystals over a wide temperature range: alkali-metal-ion-induced phase transition from discotic nematic to columnar phases. *J. Am. Chem. Soc.* **129**, 13364–13365 (2007).
